# Extra-uterine (abdominal) full term foetus in a 15-day pregnant rabbit

**DOI:** 10.1186/s12917-017-1229-7

**Published:** 2017-11-03

**Authors:** Francisco Marco-Jiménez, Ximo García-Domínguez, Jesús Valdes-Hernández, José Salvador Vicente

**Affiliations:** Instituto de Ciencia y Tecnología Animal, Universitad Politècnica de València, 46022 Valencia, Spain

**Keywords:** Asymptomatic, Abdominal pregnancy, Placenta

## Abstract

**Background:**

While ectopic pregnancies account for 1–2% of all pregnancies, abdominal pregnancy is extremely rare, accounting for approximately 1% of ectopic pregnancies. Extrauterine abdominal pregnancy is defined as the implantation and development of an embryo in the peritoneal cavity. The present report is the first of an incidental case of abdominal pregnancy within four full-term foetus simultaneously with 2 weeks of physiological gestation in a healthy doe rabbit.

**Case presentation:**

The doe was born on November 3, 2014 and the first partum took place on May 18, 2015. The doe had previously delivered and weaned an average of 12.0 ± 1.41 live kits at birth (no stillbirths were recorded) during 5 consecutive pregnancies. The last mating was on December 18, 2015 and the detection of pregnancy failure post breeding (by abdominal palpation) on December 31, 2015. Then, the doe was artificially inseminated on January 27, 2016, diagnosed pregnant on February 11, 2016 and subsequently euthanized to recover the foetus. A ventral midline incision revealed a reproductive tract with 12 implantation sites with 15 days old foetus and 4 term foetus in abdominal cavity. There were two foetus floating on either side of the abdominal cavity and two suspended near the greater curvature of the stomach. They were attached to internal organs by means of one or 2 thread-like blood vessels that linked them to the abdominal surfaces.

**Conclusions:**

In our opinion a systematic monitoring of rabbit breeding should be included to fully understand and enhance current knowledge of this phenomenon of abdominal pregnancy.

## Background

To satisfy the demand for rabbit meat, mainly in Mediterranean countries, breeders have developed an intensive and rationalized rabbit production system based on selected crossbred lines and specific reproductive management. European rabbit meat production is approximately 500 000 tons, corresponding to a 30% share of world production [1]. However, rabbits account for the second highest number of animals slaughtered per year in the European Union-27, with 326,619 × 103 head in 2010 [2]. Setting up rabbit production systems has allowed researchers to observe or describe pathologies previously unknown in non-intensive rabbit farms [3] resulting in a high replacement rate (i.e. around 10% per month, [4]). In this context, during a necropsy study of adult fertile females from two rabbit farms in Spain, 28 out of 550 rabbit does were replaced due to diagnosis of abdominal pregnancies (mummified intra- or extrauterine foetus, abscesses, pyometras, etc.) [3]. Undetected extrauterine pregnancy is frequently associated with fatal outcomes to the doe and offspring, including the formation of mummified foetus, which may eventually become calcified [3, 5]. Abdominal pregnancy specifically indicates an implantation in the peritoneal cavity and, although uncommon, has been described in several species, including rabbit [3, 5]. The objective of this case presentation was to describe an incidental found case of abdominal pregnancy with placentation in which four full-term foetus developed to term simultaneously with 2 weeks of physiological gestation in a healthy doe rabbit selected to increase litter size at weaning.

## Case presentation

This study involved a Spanish commercial rabbit line named LP (long productive), housed on the farm belonging to the Animal Science Department of the Polytechnic University of Valencia (Spain). This line was established between 2002 and 2003 by applying a very high selection intensity (i.e. two to five females were selected from 1,000) to obtain females with a long reproductive lifespan (i.e. at least 25 parturitions averaging a minimum of 7.5 live born kits per parturition) [6], which led to very robust females [7]. After its foundation (10th generation), this line was selected to increase litter size at weaning. Does were housed at the Polytechnic University of Valencia experimental farm in flat deck indoor cages (75 × 50 × 30 cm), with free access to water and commercial pelleted diets (minimum of 15 g of crude protein per kg of dry matter (DM), 15 g of crude fibre per kg of DM, and 10.2 MJ of digestible energy (DE) per kg of DM). The photoperiod was set to provide 16 h of light and 8 h of dark, and the room temperature regulated to keep temperatures between 10 and 28 °C.

Frequent health monitoring showed no microbial pathogens, endo-, or ectoparasites in the animals. The doe was born on November 3, 2014 and the first partum took place on May 18, 2015. The doe had previously delivered and weaned an average of 12.0 ± 1.41 live kits at birth (no stillbirths were recorded) during 5 consecutive pregnancies. The last mating was performed on December 18, 2015 and the detection of pregnancy failure post breeding (by abdominal palpation) on December 31, 2015. Then, the doe was artificially inseminated (AI) in January 27, 2016. No fertility drugs were used to enhance the number of pups in the litter. Receptivity of doe was determined observing the vulvar colour and turgescence, considering receptive those with red/purple and swollen vulva. AI was performed with 0.5 mL of fresh semen based on motility criteria and diluted 1:5 with tris-citric-glucose diluent [8]. Immediately after insemination, ovulation was induced by an intramuscular injection of 1 μg of Buserelin Acetate (Suprefact, Hoescht Marion Roussel, S.A., Madrid, Spain). Doe was diagnosed pregnant on February 11, 2016 and subsequently euthanized by intravenous injection of 200 mg/kg of pentobarbital sodium into the marginal ear vein (Dolethal, Vetoquinol SA, Lure, France) to recover the foetus. Foetus were used for transplantation purposes to replace the function of diseased organs [9, 10] (Marco-Jiménez et al., 2015; Garcia-Dominguez et al., 2016).

**Fig. 1:**
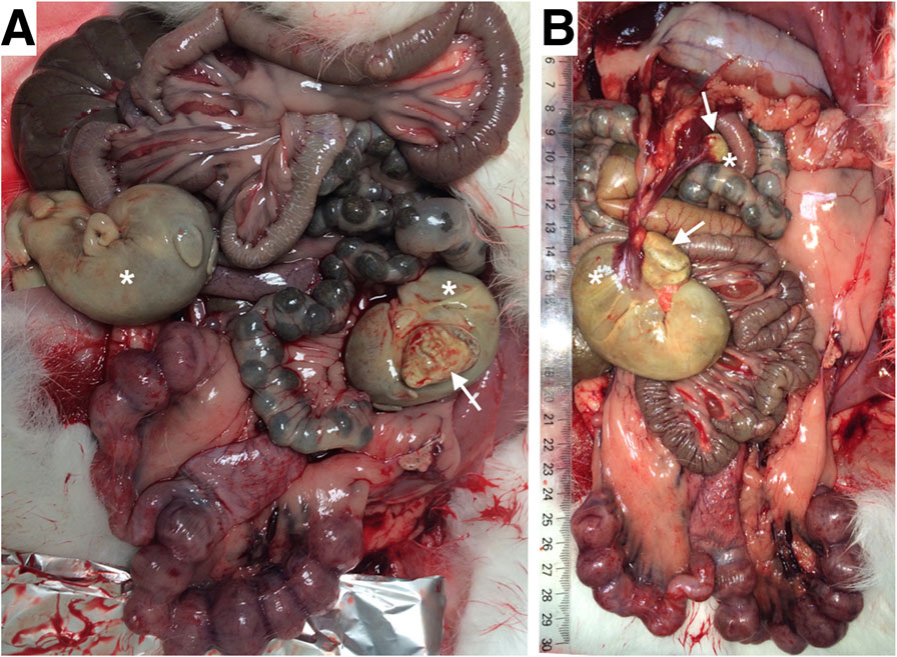
Photograph of rabbit doe without lesions in reproductive tract with abdominal pregnancy with four full-term foetus (asterisks) simultaneously with 2 weeks of physiological gestation, dorsoventral view. **a** Two free in the abdominal cavity, one showed placental attachments (arrow). **b** Two attached to the omentum near the stomach, with placental attachments identified (arrows)

**Fig. 2:**
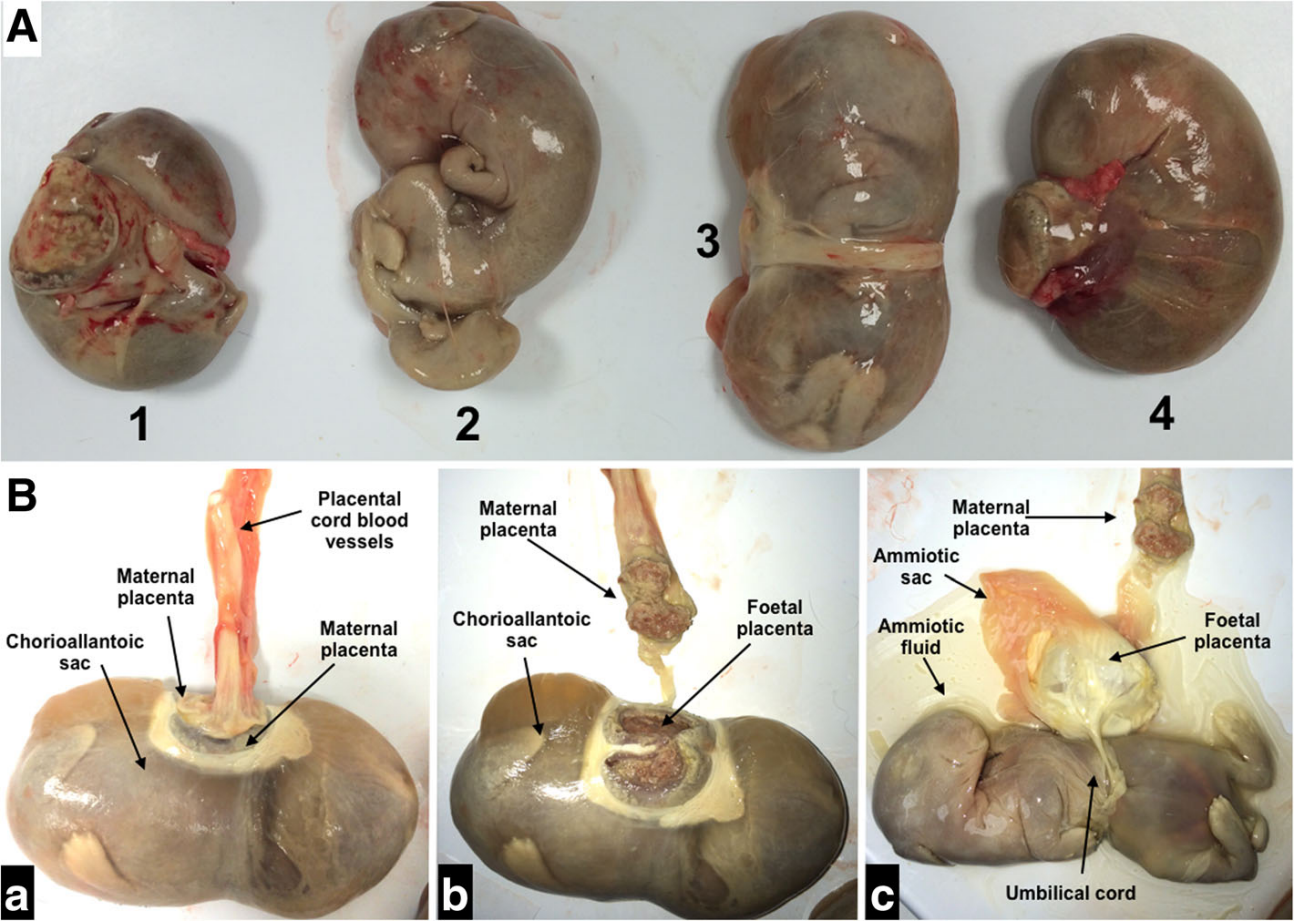
Photograph of full-term foetus recovered after abdominal gestation. (**a**) Foetus number 1, 3 and 4 present chorioallantoic placentas, whereas number 2 does not. Foetus 3 and 4 showed fluid in the chorioallantoic sac. (**b**) Detail of chorioallantoic placentas and the placental cord blood vessels (*a*). Detail of the maternal and fetal placenta (*b*). Detail of the amniotic sac and umbilical cord (*c*)

**Fig. 3:**
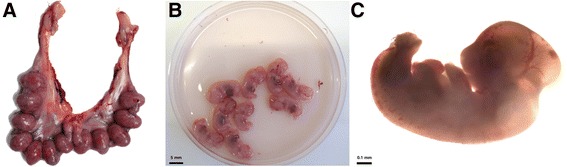
Photograph of reproductive tract and foetus at day 15 of gestation. **a** Implantation sites. **b** Recovered foetus. **c** Microscopic appearance

A ventral midline incision, xiphoid to pubis, revealed a reproductive tract with 12 implantation sites with 15 days old foetus and 4 apparently full term foetus in abdominal cavity. There were two foetus floating on either side of the abdominal cavity (Fig. 1a) and two suspended near the greater curvature of the stomach (Fig. 1b). They were attached to internal organs by means of one or 2 thread-like blood vessels that linked them to the abdominal surfaces. All the foetus were early mummified and appeared to be covered by a smooth and yellow/green serosal surface. All the masses were apparently term foetus weighing 39.5 to 63.8 g and measuring 8.5 to 11.1 cm (crown to rump, Fig. 2a). Early mummification was the hallmark of all extrauterine masses, although two of them were encapsulated by their chorioallantoic sacs and with amniotic fluid present. Dissection was performed on these two masses with removal of closely adhered remains of the allantois, which revealed fetal remnants with closed eyes, partially autolysed abdominal viscera, bone, hair, whiskers and the umbilical cord Fig. 2b). Gross examination of the doe’s reproductive system including uterus, vascular structures and ovaries revealed normal morphology (corpus luteum present), with no adhesions between the uterus and other viscera. No evidence of scar formation or recent rupture was noted. Each uterine horn was examined to detect fetal or embryonic resorption or fetal death. The foetus were dissected from the uterus and placed in fresh Dulbecco’s modified Eagle’s medium for examination under a stereomicroscope (Fig. 3). All the foetus were morphologically normal and weighed 0.49 ± 0.011 g on average.

## Discussion

Although the discovery of an ectopic foetus is often an incidental finding, as the animals may or may not display clinical signs [11], the incidence of ectopic pregnancy in humans and rabbit has increased from 0.4 to 2% and from 0.67 to 5.1%, respectively (human [12, 13] and rabbit [3]). However, whilst ectopic pregnancies account for 1–2% of all pregnancies, abdominal pregnancy is extremely rare, accounting for approximately 1% of ectopic pregnancies [14]. Moreover, a late term abdominal pregnancy with a viable foetus is an even rarer phenomenon, with few reported cases in the literature [15, 16]. The current report illustrates a case of primary form of abdominal pregnancy (when fertilization occurs outside the uterus after an oocyte is incidental released from the fimbria) within full-term foetus simultaneously with 2 weeks of physiological gestation.To the best of our knowledge, only one report has found one animal with natural gestation and abdominal pregnancy at the same time in rabbit [3], but the status of the foetus was unknown. Term pregnancies occurring subsequent to and in conjunction with extrauterine foetus are documented in other species [17, 18]. The diagnosis of an asymptomatic abdominal pregnancy is generally difficult during routine evaluation [3, 5]. As clinical signs of illness are usually absent in rabbit, abdominal pregnancies are not usually discovered. Moreover, as the method used to determine pregnancy is based on detecting the foetus in the uterus by palpating the abdomen between 10 and 14 days after mating, mass or masses (mummified intra- or extrauterine foetus, abscesses, pyometras, etc.) could be detected, but females will be replaced from the farm without a necropsy examination. More research is therefore necessary to determine if the replacement rate of females when masses are palpated is a consequence of uterine, ectopic or abdominal masses.

In rabbit females, the main productive challenges are linked to the reproductive rhythm, i.e. the intensity of reproduction timing [19] and the litter size during lactation [20]. Consequently, intensive selection of farm animals to increase productive traits has resulted in specialized breeds and strains in livestock animals [7]. One plausible explanation for the incidental observed abdominal pregnancy could be the female genotype. The LP line was constituted by selection for hyper longevity and reproductive criteria [6]. Current practices aimed at increasing the reproductive load often fail to take into consideration the effects of age, hormones, gestation overlap and parity as stressors [5]. Perhaps the high replacement rate of females in meat producing rabbits (e.g. >120%, [6]) could be related to selection for reproductive intensity. However, a broader understanding and enhanced current knowledge of this infrequent phenomenon of abdominal pregnancy is needed to increase diagnostic efficiency and precision, as well as preventive programmes aimed at preserving overall fertility.

## Conclusions

To our knowledge, this is the first reported case of abdominal pregnancy with full-term foetus simultaneously with 15 days of physiological gestation in rabbit. In our opinion a systematic monitoring of rabbit breeding should be included to fully understand and enhance current knowledge of abdominal pregnancy. Further research focusing on more population may help in better characterizing this phenomenon and its risks.
